# Single gene targeted nanopore sequencing enables simultaneous identification and antimicrobial resistance detection of sexually transmitted infections

**DOI:** 10.1371/journal.pone.0262242

**Published:** 2022-01-21

**Authors:** Liqing Zhou, Andrea Lopez Rodas, Luz Marina Llangarí, Natalia Romero Sandoval, Philip Cooper, Syed Tariq Sadiq

**Affiliations:** 1 Applied Diagnostic Research and Evaluation Unit, Institute for Infection and Immunity, St George’s, University of London, London, United Kingdom; 2 School of Medicine, Faculty of Health and Life Sciences, Universidad Internacional del Ecuador, Quito, Ecuador; 3 Grups de Recerca d’Amèrica i Àfrica Llatines, GRAAL, Barcelona, Spain; GGD Amsterdam, NETHERLANDS

## Abstract

**Objectives:**

To develop a simple DNA sequencing test for simultaneous identification and antimicrobial resistance (AMR) detection of multiple sexually transmitted infections (STIs).

**Methods:**

Real-time PCR (qPCR) was initially performed to identify *Neisseria gonorrhoeae* (NG), *Chlamydia trachomatis* (CT), *Mycoplasma genitalium* (MG) and *Trichomonas vaginalis* (TV) infections among a total of 200 vulvo-vaginal swab samples from female sex workers in Ecuador. qPCR positive samples plus qPCR negative controls for these STIs were subjected to single gene targeted PCR MinION-nanopore sequencing using the smartphone operated MinIT.

**Results:**

Among 200 vulvo-vaginal swab samples 43 were qPCR positive for at least one of the STIs. Single gene targeted nanopore sequencing generally yielded higher pathogen specific read counts in qPCR positive samples than qPCR negative controls. Of the 26 CT, NG or MG infections identified by qPCR, 25 were clearly distinguishable from qPCR negative controls by read count. Discrimination of TV qPCR positives from qPCR negative controls was poorer as many had low pathogen loads (qPCR cycle threshold >35) which produced few specific reads. Real-time AMR profiling revealed that 3/3 NG samples identified had *gyrA* mutations associated with fluoroquinolone resistance, 2/10 of TV had mutations related to metronidazole resistance, while none of the MG samples possessed *23S rRNA* gene mutations contributing to macrolide resistance.

**Conclusions:**

Single gene targeted nanopore sequencing for diagnosing and simultaneously identifying key antimicrobial resistance markers for four common genital STIs shows promise. Further work to optimise accuracy, reduce costs and improve speed may allow sustainable approaches for managing STIs and emerging AMR in resource poor and laboratory limited settings.

## Introduction

Sexually transmitted infections (STIs) remain a major public health problem worldwide, with an estimated 357 million new cases of *Chlamydia trachomatis* (CT), *Neisseria gonorrhoeae* (NG), *Treponema pallidum* (TP) and *Trichomonas vaginalis* (TV) per year [1]. High rates of the STI, *Mycoplasma genitalium* (MG), have also been reported worldwide [2] and is associated with genital discharge syndrome in men and reproductive sequelae in women [3]. All these STIs are generally curable with existing, effective single-dose antibiotic regimens but, if left undiagnosed and/or untreated, can result in serious long-term reproductive health sequelae, particularly for women. Antimicrobial resistance (AMR) among some STIs to multiple classes of antibiotics has spread rapidly in recent years. For NG, loss of extended spectrum cephalosporins as first line empirical treatment is a major concern [4] as circulating multi- and extensive-drug resistance clones have been detected internationally [5]. For MG, macrolide resistance is now widely but not universally reported with increasing rates of resistance to fluoroquinolones also detected [6]. These developments have made treatment, and particularly empirical treatment challenging.

New World Health Organization (WHO) guidelines reinforce the need to treat these STIs with the right antibiotic, at the right dose, and the right time to reduce spread and improve sexual and reproductive health. Laboratory based nucleic-acid amplification tests (NAAT) for detection of NG, CT, TV and MG are well established using various gene targets, for example, GenoQuick^®^ CT (Hain Lifescience, Germany) which specifically and simultaneously detects both the gene encoding outer membrane protein 1 (omp-1) and the cryptic plasmid. For both NG and MG, accurate and rapid diagnostics which also predict antibiotic susceptibility are likely to be needed to achieve this. Laboratory based NAAT for detection of NG, CT, TV and MG are the current gold standard for detection, providing high sensitivity and specificity. NAAT are widely used in high-income countries but are often unavailable in resource-poor settings. Culture-based antimicrobial susceptibility testing (AST) for NG remains gold standard for phenotypically predicting AMR and commonly takes usually two to five days to obtain a result in UK microbiology laboratories (personal communication, Sadiq). This in practical terms may be too late to initiate targeted antibiotic therapy, particularly for hard to reach vulnerable populations. MG is difficult to cultivate and requires cell culture, not usually feasible in most clinical settings.

Advances in understanding AMR in NG, MG and TV have allowed for development of NAAT- based AMR detection. For example, absence of *gyrA* mutations at amino acid positions S91 and D95 of NG accurately predicts fluoroquinolone susceptibility [7, 8], presence of mutations at positions 2058 and 2059 (*Escherichia coli* numbering) in region V of the 23S rRNA gene in MG is associated with failure of treatment with azithromycin [9–11], and a single nucleotide polymorphism (SNP) in *ntr6* gene (ntr6 A238T) of TV is associated with metronidazole resistance, which may have diagnostic value for metronidazole resistance [12]. Use of NAAT-based AMR tests, however, may have limitations due to continually changing mutations and novel mechanisms of resistance evolving under ongoing treatment selection pressures. Whole genome sequencing (WGS) using high throughput sequencing platforms enables detection of multiple genes/pathogens in a single run, which may address this challenge to some degree and also give added value in identifying phylogenetic relationships in identified infections [13, 14]. However, for diagnostic purposes WGS itself can be constrained by sample preparation, cost etc., making it unsuitable for near patient applications.

Oxford Nanopore Technologies’ (ONT) portable MinION DNA sequencer, together with its recent MinIT hand-held processor, may offer advantages as an accurate diagnostic in resource-limited settings. Herein we report early phase study among female sex workers (FSWs) in Ecuador, of using the MinION, controlled by a smartphone-operated MinIT to detect the four common STIs (NG, MG, TV and CT) and profile AMR to ciprofloxacin in NG, azithromycin in MG and metronidazole in TV utilising a single gene targeted approach.

## Materials and methods

### Ethics statement

Written and verbal informed consent was obtained from FSW participants all of whom were 18 years or older and none of whom received any compensation for their participation. The study was conducted according to the Declaration of Helsinki and approved by the Ethical Committee of Universidad Internacional del Ecuador (02-02-17).

### Clinical sample collection and DNA preparation

A cross-sectional study of STIs was conducted among FSWs in a primary health centre in Quito, Ecuador, during the last quarter of 2017 [15]. Vulvo-vaginal swab samples were collected by clinicians using Xpert^®^ CT/NG Patient-Collected Vaginal Swab Specimen Collection Kit (Cepheid). DNA from swab samples was prepared using PureLink Genomic DNA Mini Kit (Thermo Fisher Scientific) according to the manufacturer’s instructions and quantified using a NanoDrop Spectrometer. STI identification of swab samples was performed by singleplex qPCR for individual STI pathogen. The DNA samples were stored at -20 °C till the present study.

### STI identification of swab samples by qPCR

Swab samples were initially screened by singleplex qPCR to identify NG, TV, MG and CT infections, with well-studied gene targets and primers, as shown in Table 1. qPCR was performed using Applied Biosystems 7500 Fast Real-Time PCR System in a volume of 10 μl containing 5 μl of TaqMan^™^ Fast Universal PCR Master Mix, 1 μl of 10x Exogenous Internal Positive Control (IPC) Mix, 0.2 μl of 50x IPC DNA, 250 nM each of the primers, 100 nM each of the probes and 50 ng of template DNA. Cycling parameters were 95°C for 10 min, followed by 40 cycles of 95°C for 15 sec, 60°C for 1 min. Positive STI samples and some negatives identified by qPCR were used for this study.

**Table 1:** Gene targets, primers and probes used in this study.

**a) Initial real-time PCR (qPCR) for STI testing on samples**
*Trichomonas vaginalis*: *T*. *vaginalis* specific repeat DNA fragment (amplicon 92 bp) [16]
TV_F: AAAGATGGGTGTTTTAAGCTAGATAAGG
TV_R: TCTGTGCCGTCTTCAAGTATGC
TV_P: [6FAM]AGTTCATGTCCTCTCCAAGCGTAAGT[BHQ1]
*Mycoplasma genitalium*: MgPa gene (amplicon 78 bp) [17]
MG_F: GAGAAATACCTTGATGGTCAGCAA
MG_R: GTTAATATCATATAAAGCTCTACCGTTGTTATC
MG_P: [6FAM]ACTTTGCAATCAGAAGGT[MGBNFQ]
*Chlamydia trachomatis*: The cryptic plasmid (amplicon 71 bp) [18]
CT_F: CATGAAAACTCGTTCCGAAATAGAA
CT_R: TCAGAGCTTTACCTAACAACGCATA
CT_P: [6FAM]TCGCATGCAAGATATCGA[MGBNFQ]
*Neisseria gonorrhoeae*: opa (amplicon 90 bp) [19]
NG_F: TTGAAACACCGCCCGGAA
NG_R: TTTCGGCTCCTTATTCGGTTTGA
NG_P: [6FAM]CCGATATAATCCGCCCTTCAACATCAG[BHQ1]
**b) Targeted PCR (tPCR) for nanopore sequencing** *
*Trichomonas vaginalis*: Nitroreductase family protein (*ntr6*; amplicon 746 bp)
TV_NTR6_BC_F:
5’TTTCTGTTGGTGCTGATATTGCCTTCATTGAATTTATTCGTTCAAAATT
TV_NTR6_BC_R:
5’ACTTGCCTGTCGCTCTATCTTCTTATTCAATGTATGTAACCTTTCTAA
*Mycoplasma genitalium*: 23S ribosomal RNA (amplicon 688 bp)
Mg 23S_BC_1992F:
5’ TTTCTGTTGGTGCTGATATTGCCCATCTCTTGACTGTCTCGG
Mg 23S_BC_2679R:
5’ ACTTGCCTGTCGCTCTATCTTCTCCTCTCGTACTAGAAGCAAAG
*Chlamydia trachomatis*: Major outer membrane protein 1 (*omp1*; amplicon 1060 bp)
CT_OMP1_BC_F:
5’ TTTCTGTTGGTGCTGATATTGCTTTGCCGCTTTGAGTTCTGCT
CT_OMP1_BC_R:
5’ ACTTGCCTGTCGCTCTATCTTCCAATACCGCAAGATTTTCTAGATTTC
*Neisseria gonorrhoeae*: *gyrA* (amplicon 1181 bp)
NG_gyrA_BC_F:
5’ TTTCTGTTGGTGCTGATATTGCATCCGCCACGACCACAAATT
NG_gyrA_BC_R:
5’ ACTTGCCTGTCGCTCTATCTTCATATTGGACAGTGCGACGGC

*developed in this study.

### Targeted PCR (tPCR) amplification and barcoding

Both qPCR positive and negative samples for NG, MG, TV and CT plus an NG positive control were amplified by tPCR, targeting genes *gyrA* (NG), *23S rRNA* (MG), *ntr6* (TV) and *omp1* (CT) with the primers listed in Table 1. tPCR reactions for each target were performed in a 50 μl volume consisting of 25 μl LongAmp^®^ Taq 2X Master Mix (NEB), 200 nM each of tPCR primers and 100 ng DNA template as follows: 95 °C for 3 min followed by 35 cycles of 95 °C for 30 sec, 58 °C for 30 sec, 65 °C for 1 min 30 sec, and one cycle of 65 °C for 5 min. tPCR amplicons were purified and subjected to a second PCR for barcoding using PCR Barcoding Expansion 1–96 (EXP-PBC096, ONT) according to the manufacturer’s instructions. In this study, we used a sequence-based method for pathogen detection by mapping a ‘unique’ gene sequence of pathogens of interest to a reference database, and therefore specificity of detection lies within the sequence itself. This is different from conventional nucleic acid amplification methods, in which primer specificity is crucial to detection accuracy. The AMR-associated gene targets we used have a unique nucleotide sequence specific to pathogen of interest and can differentiate itself from other species. As a general practice we have searched nucleotide databases by BLAST when designing tPCR primers in order to discriminate as much as possible the target from other closely related bacterial species. Furthermore, to enhance the read counts of pathogen of interest we selectively designed the tPCR primers with some mismatch base(s) to other closely related bacterial species at the 3’ end for preferential target amplification.

### Nanopore sequencing and data analysis

A DNA sequencing library was constructed from a pool of barcoded tPCR amplicons of different clinical samples using Ligation Sequencing Kit (SQK-LSK108) and sequenced using FLO-MIN106 R9 Version Flow Cell MK I Spot-ON on portable DNA sequencer MinION MK I which was controlled by a smartphone operated MinIT (ONT) according to the manufacturer’s instructions. Sequencing data were uploaded onto Metrichor Epi2ME (ONT) and analyzed using the workflow Fastq Antimicrobial Resistance r3.3.2 which included three components: QC and Barcoding [rev. 3.10.4], WIMP [rev. 3.4.0] and ARMA CARD [rev. 1.1.6]. QC and Barcoding component contains quality score (qscore) and barcode filter, cutting off reads with a qscore below the threshold (min qscore:7) which were uploaded for analysis, and demultiplexing barcodes if the sequenced samples were barcoded. WIMP (what’s In My Pot) allows for species identification by classifying read sequences against the standard Centrifuge database (including RefSeq complete genomes for bacteria). ARMA (antimicrobial resistance mapping application) performs antimicrobial resistance identification by aligning input reads with minmap2 against all reference sequences available in the CARD database (CARD version 1.1.3). TV, an anaerobic, flagellated protozoan parasite, was not included in RefSeq for bacteria and therefore a TV G3 nitroreductase family protein (TVAG_354010, ntr6) reference was uploaded using Fasta Reference Upload r3.2.2 and TV identification was performed using Fastq Custom Alignment r3.2.2 against the *ntr6* reference sequence. Read counts were log-transformed and compared between qPCR positive and negative samples by t-test.

### BLAST search

DNA sequences of NG, MG and TV reads were extracted from read FASTQ files and BLAST-aligned against the reference sequences: *Neisseria gonorrhoeae* FA 1090 *gyrA* reference (NC_002946.2:c621189-618439), *Mycoplasma genitalium* strain G-37 *23S rRNA* gene (NR_077054.1) and *Trichomonas vaginalis* G3 nitroreductase family protein reference (TVAG_354010) respectively. Part of the aligned sequences flanking AMR-associated mutations were used to construct a consensus sequence using MultAlin [20]. AMR-associated mutations were confirmed by manually comparing the consensus sequence against the reference.

## Results

### STI identification of swab samples by qPCR

A total of 200 vulvo-vaginal swab samples from FSWs were initially screened by qPCR, and 43 were found positive, including 37 single infections (11 MG, 19 TV and 7 CT) and six co-infections (two CT/TV and one each of CT/NG, NG/TV, CT/NG/TV and CT/TV/MG), as shown in Table 2.

**Table 2:** STI identification of swab samples by qPCR and nanopore sequencing.

Sample ID	NG	CT	TV	MG	Ct value	Read counts				
						Analysed	NG	CT	TV	MG
60	-	-	-	+	32.505	784	1	0	1	91
166	-	-	-	+	30.436	25188	2	0	9	19968
85	-	-	-	+	35.403	1931	1	3	4	477
31	-	-	-	+	34.976	7988	0	2	4	1416
230	-	-	-	+	28.800	23263	0	2	5	18676
103	-	-	-	+	33.375	18383	3	0	5	7262
13	-	-	-	+	32.980	26039	0	0	9	20902
109	-	-	-	+	29.870	39248	2	0	7	39248
239	-	-	-	+	36.210	3133	1	0	5	250
69	-	-	-	+	35.810	3412	0	0	9	737
263	-	-	-	+	29.592	2446	1	0	2	1457
260^a^	-			+	36.672	633	1	1	4	1
51	-	-	-	-		2952	2	1	1	4
75	-	-	-	-		589	0	2	4	2
77	-	-	-	-		252	2	0	0	1
24	-	-	+	-	20.607	20815	3	0	10368	2
58	-	-	+	-	25.250	23714	3	0	17712	2
235	-	-	+	-	36.170	29942	1	3	5	4
256	-	-	+	-	39.995	25061	2	1	8	2
146	-	-	+	-	39.695	68032	3	0	8	4
144	-	-	+	-	24.409	22359	1	2	6026	0
145	-	-	+	-	38.037	32349	2	0	10	4
261	-	-	+	-	38.596	37283	2	0	8	6
90	-	-	+	-	23.581	17230	2	1	9824	4
106	-	-	+	-	39.839	18291	0	2	8	2
92	-	-	+	-	40.00	19009	1	0	1	0
72	-	-	+	-	36.897	20163	2	0	11	2
140	-	-	+	-	35.249	22397	1	0	6	6
253	-	-	+	-	37.778	30217	1	0	5	5
41	-	-	+	-	38.867	10297	2	2	7	11
124	-	-	+	-	27.540	23714	2	1	17882	7
71	-	-	+	-	27.565	4949	2	1	2101	5
258	-	-	+	-	38.636	14925		10	17	5
105	-	-	+	-	30.327	9029	4	2	1937	8
221^b^	-		+	-	38.171	2513	1	1	6	2
89^b^	-		+	-	35.079	30401	2	0	7	5
91^c^		-	+	-	21.186	29511	1	1	16907	2
39^d^			+	-	21.381	41735	1	0	41486	0
260^a^	-		+		27.556	35209	0	1	15706	0
79	-	-	-	-		28262	1	0	12	1
84	-	-	-	-		6617	3	0	8	4
98	-	-	-	-		21575	3	0	7	4
260^a^	-	+			35.905	17328	5	82	3	6
94	-	+	-	-	29.812	12745	3	530	4	3
259	-	+	-	-	30.331	8309	2	672	8	6
242	-	+	-	-	31.946	8443	1	1840	10	3
236	-	+	-	-	35.936	10335	3	65	7	8
157	-	+	-	-	30.126	10277	1	25	9	4
114	-	+	-	-	30.464	5086	1	2317	6	1
6	-	+	-	-	31.232	7582	1	127	18	24
221^b^	-	+		-	35.968	2978	2	72	4	3
89^b^	-	+		-	29.932	6565	0	220	10	3
30^e^		+	-	-	30.886	14878	2	13796	3	1
39^d^		+		-	30.536	999	1	797	3	1
102	-	-	-	-		3095	0	7	5	1
107	-	-	-	-		4664	2	1	7	2
125	-	-	-	-		101	1	0	9	2
30^e^	+		-	-	19.033	27210	21087	0	1	0
91^c^	+	-		-	20.035	39223	36590	1	5	0
39^d^	+			-	21.504	28317	12325	3	3	0
126	-	-	-	-		14121	2	0	2	1
189	-	-	-	-		9168	3	1	1	5
193	-	-	-	-		11334	1	0	3	2
NG positive control						7937	7659	0	1	0

^a^ CT, TV and MG mixed infection.

^b^ CT and TV mixed infection.

^c^ NG and TV mixed infection.

^d^ NG, CT and TV mixed infection.

^e^ Ng and CT mixed infection.

### STI identification and AMR detection by single gene tPCR nanopore sequencing

All 43 qPCR positives plus 12 negatives (3 for each pathogen) and an NG positive control were subjected to single gene tPCR nanopore sequencing in one barcoded sequencing library. A sequencing run of 10 hours produced 1,499,872 reads of which 1,127,703 (76%) reads passed QC, with total yield of 1.2 gigabases, average qscore 8.34 (S1 Fig) and average sequence length 816 bases. To identify MG, NG and CT, all reads (1,127,703) which passed QC were analyzed by WIMP, resulting in 810,683 (72%) classified reads of which 43,491 (5.4%) were non-barcoded. For TV identification, Fastq Custom Alignment workflow produced 148,458/1,127,703 (13%) read alignments with TV *ntr6* sequence of which 66,500 (5.9%) were non-barcoded.

**Fig 1:**
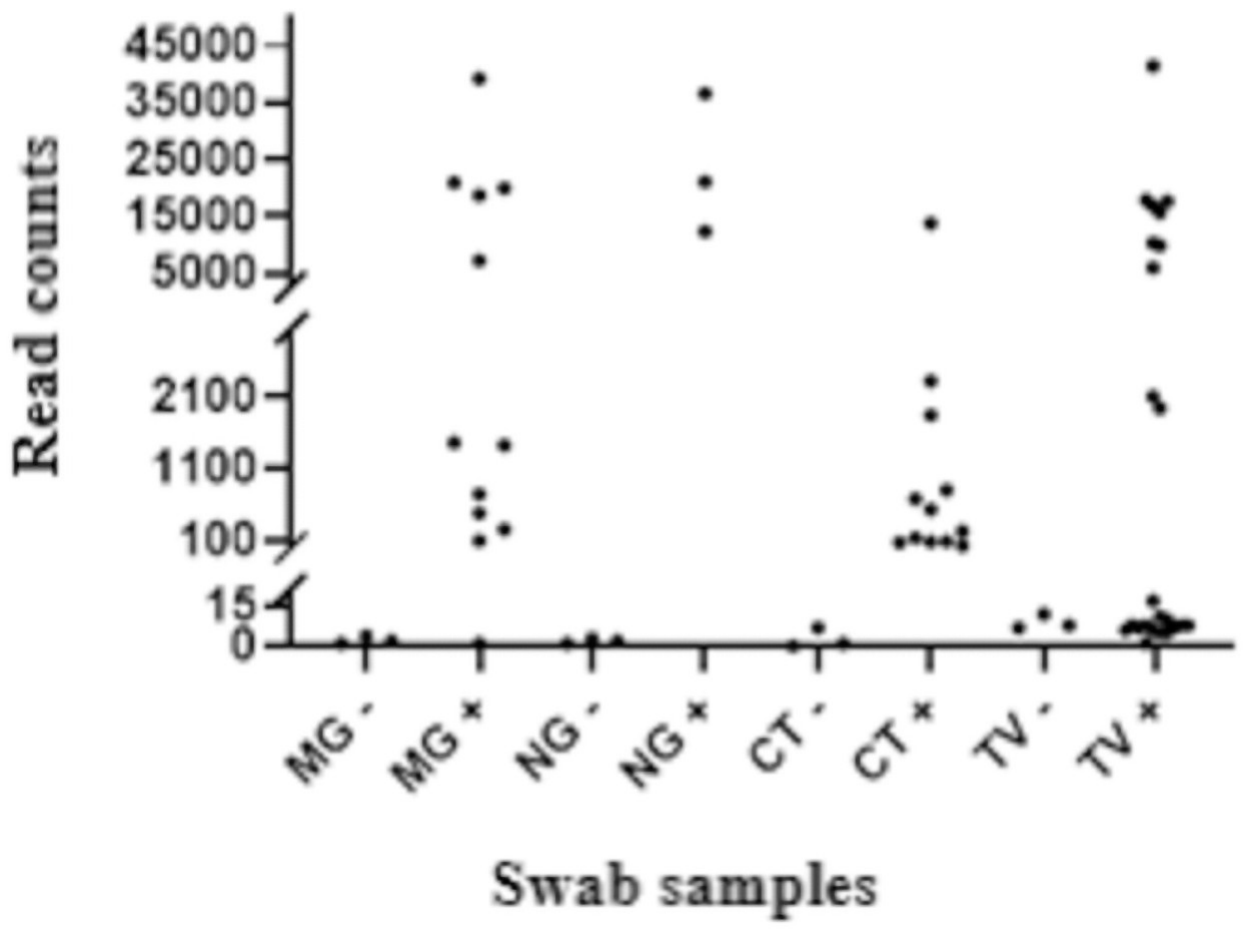
Pathogen read counts in the qPCR positive and negative STI samples analyzed by single gene targeted PCR nanopore sequencing.

Targeted amplicon sequencing from clinical samples, as expected, still non-specifically produced some reads from other microbial and/or host DNA in the sample. Among 810,683 classified reads of the whole sequencing library, 560,601 (69%) mapped to the human genome. Details on the bacterial species that was mapped are shown in S1 Table. The ranges of classified reads mapped to the pathogens of interest are 0.5–95% (median 9.6%) for the CT positive samples, 0–86.6% (median 48.5%) for the MG positive samples and 92.3–95% (median 94.7%) for the NG positive samples, while the range of analysed reads mapped to TV in the TV positive samples is 0–99.4% (median 0.1%). However, for STI pathogens, qPCR positive samples generally yielded higher specific read counts than qPCR negative controls, all of the latter of which had fewer than 15 reads (Fig 1). Mean log_10_ read counts were higher in qPCR positive NG, MG and CT samples compared to negative by t-test (S2 Table) but not significantly for TV as 14/24 TV read counts in TV qPCR positive samples were very low (<20 reads). NG, MG and CT read counts clearly separated qPCR positive and negative samples except for one MG qPCR positive sample that had an absolute read count of 1 (Fig 1). The samples having <20 reads generally had a low load of pathogen, as evidenced by qPCR cycle threshold (Ct) being >35 (Table 2; S2 Fig).

The ARMA workflow assigned 86,430/810,683 (11%) classified reads to antibiotic resistance genes in the CARD database, >99.99% of which were identified to be fluoroquinolone resistant *gyrA* gene in NG, using protein variant model. This analysis indicated that 3/3 NG clinical strains identified were fluoroquinolone-resistant, while none of the MG strains were resistant to macrolide antibiotics. The NG positive control, phenotypically susceptible to fluoroquinolones, and genotypically wild-type by BLAST (see below), was mis-identified to be fluoroquinolone-resistant. TV antimicrobial resistance was not profiled by ARMA due to the absence of TV *ntr6* entries in the databases. Instead, it was analyzed by manual BLAST search against TV G3 ntr6 reference sequence (see below).

### AMR confirmation by manual BLAST search

A manual BLAST search was performed to ascertain the accuracy of the pathogen AMR identified by the ARMA workflow. The BLAST confirmed, as shown in Table 3, that all (3/3) of NG had fluoroquinolone-resistant mutations, 2/10 (20%) of TV had mutations related to metronidazole resistance, and none of MG had macrolide resistance-associated mutations. It also showed that the gyrA of NG positive control did not possess fluoroquinolone-resistant mutations.

**Table 3:** BLAST confirmation of antimicrobial resistance associated mutations identified by single gene targeted PCR nanopore sequencing*.

Bacterial gene	Sample	Antimicrobial resistance region: nucleotide sequence (NS) and amino acid (AA) change
*Neisseria gonorrhoeae*: *gyrA*	Wild type	NS: -T**C**CGCAGTTTACG**A**C-
		AA: S91 D95
	All NG samples	NS -T**T**CGCAGTTTACG**C**C-
		AA change: S91F D95A
	Positive control	NS: -T**C**CGCAGTTTACG**A**C-
		AA: S91 D95
*Mycoplasma genitalium*: 23S rRNA gene	Wild type	NS: -CGGGACGG**AA**AGACC-
		A2058, A2059 (Escherichia coli numbering)
	All MG samples	NS: -CGGGACGG**AA**AGACC-
		A2058, A2059 (Escherichia coli numbering)
*Trichomonas vaginalis*: *ntr6*	Wild type	NS: -AATGCAA**A**AGCAGAC-
		AA: K80
	2 TV samples	NS: -AATGCAA**T**AGCAGAC-
		AA change: K80STOP
	8 TV samples	NS: -AATGCAA**A**AGCAGAC-
		AA: K80

*One or more changes of the bold bases in the wild type cause antimicrobial resistance of strains.

## Discussion

We demonstrated the potential to test four common genital STIs and simultaneously detect AMR in three of them among vulnerable FSWs in Quito, Ecuador using the portable MinION DNA sequencer, controlled by smartphone, through a single gene targeted approach.

Single gene approaches for detection of infection, such as 16S rRNA sequencing have been increasingly described [21, 22], particularly with the advent of long read sequencing such as PacBio and ONT [23], which has greater likelihood of taxonomically identifying organisms at species level. We intentionally evaluated the accuracy of sequencing of a single AMR gene, as opposed to a two-stage gene approach [24], to both diagnose infection and predict AMR simultaneously in order to evaluate potential for use in resource poor field settings where simplicity and cost become increasingly important factors to consider.

Our approach appeared to have some advantages over NAAT. We demonstrated initially that genes responsible for AMR have potential to be as useful as 16S rRNA genes for diagnosis and thus may serve as a ‘two-in-one’ target. We chose for both pathogen and AMR detection the NG *gyrA*, MG *23S rRNA* and TV *ntr6*, all gene mutations in which have been shown to be predictive of AMR [7–12, 25, 26], and CT *omp-1* for CT detection [27]. As sequencing allows for identification of evolving mutations in the same genes without changing the test, our approach will therefore have value as it is adapted to other gene targets undergoing continuous evolution under selection pressure such as penA in which changes may give rise to penicillin and extended spectrum cephalosporin resistance [28]. The approach can also allow for sequencing multiple genes simultaneously alongside vigilance over any changing nature of phenotypic to genotypic association for antibiotics where AMR involves multiple mechanisms.

Clinical STI samples contain a wide range of pathogen loads being drawn frequently from largely asymptomatic patients as in the case of the FSW cohort in this study. We found that except for TV, diagnosis of NG, CT and MG by the single gene targeted nanopore sequencing appeared correct as almost NG, CT and MG positive samples had high pathogen loads. Due to the small sample size used in this study we did not determine a cut-off value of read counts for a positive STI test, but even so, our single gene approach correctly identified the majority of STI infections in the samples which had qPCR Ct<35 (higher pathogen loads) by their distinctive read counts. In the group with lower pathogen loads (Ct>35), tPCR amplification appeared not to enhance the absolute read counts of pathogen targets, possibly due to low tPCR efficiency caused by the presence of a large quantity of background DNA and non-optimal tPCR conditions, resulting in the ‘false-negatives’ observed for TV. Further optimization of tPCR conditions, for example enrichment of pathogen DNA during sample DNA preparation, may maximize the diagnostic sensitivity for this group of samples.

Regarding the Fastq Antimicrobial Resistance workflow used in this study, the component WIMP correctly identified each pathogen of interest, but we found the component ARMA CARD appeared to mis-profile the AMR of the NG control strain. This might be partially because the ARMA CARD used individual reads for matching AMR associated mutations, supported with the result obtained for the NG control strain by the manual BLAST search using the consensus sequence. A mixed AMR profile may exist in the community of pathogens of clinical samples, and therefore targeted amplification of the AMR associated genes can lead to a mixture of different AMR alleles. Therefore, any further development of ARMA, or a separate workflow, which contains a method to detect and quantify different AMR alleles, would be more appropriate for accurate prediction of AMR.

This study had some limitations. Firstly, there was a small sample size, which impacted particularly on measures of specificity. Secondly no further confirmatory sequencing was performed to confirm the mutations present, largely due to the local regulations which prevented transporting material out of Ecuador. Thus, this study should be regarded as a proof of concept study. Future work is required on larger sample sets with composite reference standards in a formal diagnostic evaluation. Additionally, we used older generation of reagents LSK-108 and tools (MinIt) in this study, which potentially impacted the output and speed of the workflow. Optimisation of amplification and target multiplexing will also be important.

Nanopore sequencing with MinION requires substantially lower infrastructure and startup cost compared to other high throughput sequencing platforms. Although arguably consumables are not optimally priced for resource poor settings, the promise of combining automated library preparations and use of disposable flow-cells has potential to use nanopore sequencing for field diagnostics. We recently demonstrated that even in developed settings implementation of rapid ciprofloxacin NAAT resistance tests for NG still requires net investment [29]. More recently ONT has developed a tablet version, MinION^™^ Mk1C which enables full sequencing and analysis to be performed in the lab and field without need for internet connection, and released rapid barcoding kits and flongle for sequencing. This development could reduce cost and improve speed further to use nanopore sequencing as a diagnostic tool. However, this will be formally evaluated in a large-scale clinical study.

In conclusion, this study demonstrated that single gene targeted nanopore sequencing for diagnosing and simultaneously identifying key antimicrobial resistance markers for four common STIs shows promise. Further work to optimise accuracy, reduce costs and improve speed may allow sustainable approaches for managing STIs and emerging AMR in resource poor and laboratory limited settings.

## Supporting information

S1 Fig Quality scores of reads produced by single gene targeted PCR nanopore sequencing. (TIF)

S2 Fig Relationship between Ct and read counts. (TIF)

S1 Table Classified species read counts and percentage of whole sequencing library and individual samples. (XLSX)

S2 Table Mean log(10) read counts in clinical swab samples analysed by single gene targeted PCR nanopore sequencing by t-test. (DOCX)
