# Supplementary figures and images for: Single gene targeted nanopore sequencing enables simultaneous identification and antimicrobial resistance detection of sexually transmitted infections
Source: PLoS One. 2022 Jan 21;17(1):e0262242. doi: 10.1371/journal.pone.0262242 (PMC8782522; doi:10.1371/journal.pone.0262242)

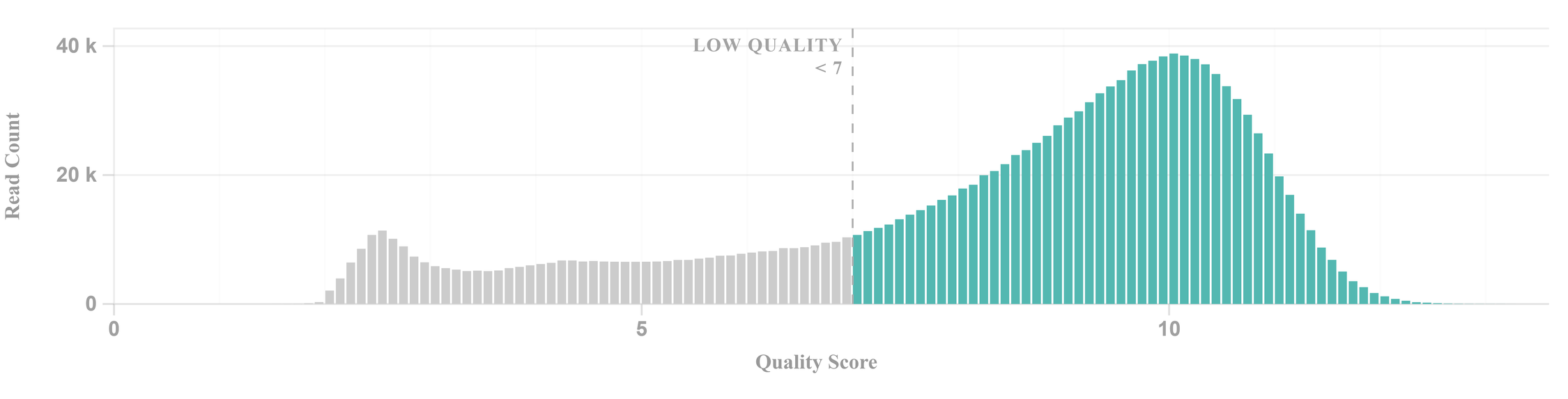

Supplement: S1 Fig — (TIF) [file pone.0262242.s001.tif]

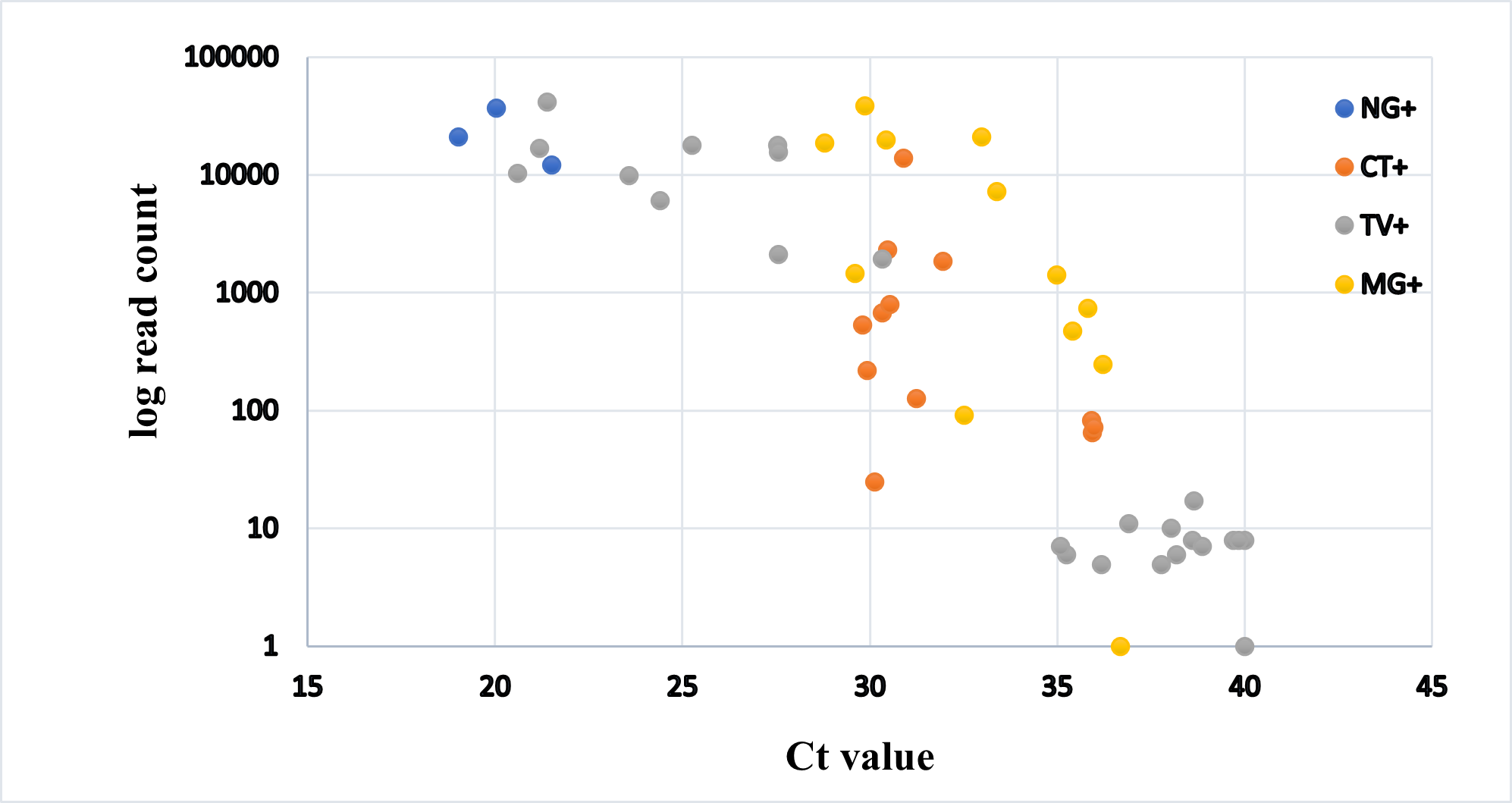

Supplement: S2 Fig — (TIF) [file pone.0262242.s002.tif]
