# Supplementary material for: Single gene targeted nanopore sequencing enables simultaneous identification and antimicrobial resistance detection of sexually transmitted infections
Source: PLoS One. 2022 Jan 21;17(1):e0262242. doi: 10.1371/journal.pone.0262242 (PMC8782522; doi:10.1371/journal.pone.0262242)
Supplement: S2 Table — (DOCX) [file pone.0262242.s004.docx]

**S2 Table. Mean log(10) read counts in clinical swab samples analysed by single gene targeted PCR nanopore sequencing by t-test**

| Pathogen | Mean Log Positive | Mean Log Control | t | p |
| --- | --- | --- | --- | --- |
| CT | 2.56 | 0.40 | 4.46 | 0.0006 |
| MG | 3.16 | 0.49 | 3.61 | 0.003 |
| NG | 4.33 | 0.46 | 23.87 | <0.0001 |
| TV | 2.19 | 0.99 | 1.28 | 0.21 |
